# Role of steroids in conservative treatment of parapharyngeal and retropharyngeal abscess in children

**DOI:** 10.1007/s00405-022-07423-6

**Published:** 2022-06-29

**Authors:** Eva Villanueva-Fernández, R. Casanueva-Muruáis, A. Vivanco-Allende, J. L. Llorente, A. Coca-Pelaz

**Affiliations:** 1 Department of Otorhinolaryngology, Hospital Universitario Central de Asturias, Avenida de Roma s/n, 3301 Oviedo, Spain; 2 Department of Pediatrics, Hospital Universitario Central de Asturias, Oviedo, Spain; 3 University of Oviedo, Instituto de Investigación Sanitaria del Principado de Asturias, IUOPA, CIBERONC, Oviedo, Spain

**Keywords:** Deep neck space infections, Retropharyngeal abscess, Parapharyngeal abscess, Antibiotic treatment, Children

## Abstract

**Purpose:**

To characterize the clinical features and outcomes of pediatric patients with retropharyngeal (RPA) or parapharyngeal abscesses (PPA) managed only with medical treatment and showing the importance of early symptoms and imaging studies in the diagnosis of deep neck space infections (DNIs) in children.

**Methods:**

A retrospective analysis of all patients diagnosed with RPA and PPA between 2007 and 2017 was performed in Hospital Universitario Central de Asturias.

**Results:**

30 children were identified, with 11 RPA and 19 PPA. 23 children (76.7%) were under 5 years old, and all were treated with intravenous amoxicillin/clavulanic acid and corticosteroids. Torticollis and fever were present in all patients. The mean length of hospital stay was 7.5 days. There were no complications associated.

**Conclusion:**

DNIs can be treated in a conservative way, reserving the surgical drainage for cases with a complication associated (airway compromise, lack of response to antibiotic therapy, immunocompromised patients). Treatment with intravenous antibiotics and corticosteroids is a safe option, reducing the duration of symptoms and the length of hospital stay.

## Introduction

Deep neck space infections (DNIs) such as retropharyngeal (RPA) and parapharyngeal abscesses (PPA) are significant clinical entity in the pediatric population. They are usually developed as complication of incompletely treated infections of the nasopharynx or oropharynx that spread to deep neck space because of developmental aspects of the neck lymphatic system [1–3]. Historically, these infections had caused significant morbidity and mortality; they can cause life-threatening complications like airway compromise, mediastinitis, internal jugular vein thrombophlebitis, pulmonary embolism, aneurysm or dissection of the internal carotid artery, dysfunction of cranial nerves IX to XII or of the sympathetic chain and sepsis [1, 4, 5]. There has been a concern that the incidence of these infections is increasing [6]. Widespread use of antibiotics in the primary care has contributed to an increase in drug-resistant bacterial strains in normal oropharyngeal flora; methicillin-resistant *Staphylococcus aureus* (MRSA) is becoming a more common organism isolated in cultures [7–9]; however, *Staphylococcus aureus* continues to be the most frequent organism isolated from pediatric neck abscesses [10]. Early diagnosis based on clinical suspicion and supportive imaging studies with computed tomography (CT) is necessary to prevent the development of these processes [1, 2]. The signs and symptoms of DNIs include fever, sore throat, neck swelling, neck pain, torticollis, drooling, trismus and even stridor [2–4]. In spite of plain radiographs can show widened prevertebral soft tissues on lateral view of the neck, nowadays CT is preferred [1, 2]. Traditionally, the recommended treatment of these infections has been surgical drainage in pus collection; however, in the last two decades, several reports have recommended conservative treatment with no surgical drainage [1–4, 6, 11]. The aim of this study is to characterize the clinical features and outcomes of patients managed only with medical treatment and showing the importance of early symptoms and imaging studies in the diagnosis of DNIs in children.

## Materials and methods

A review of the medical records of 30 patients medically treated at the Hospital Universitario Central de Asturias (Asturias, Spain) from November 2007 to November 2017 was done. 7 patients of this study were included in a previously published study [1]. Patients with symptoms indicating an inflammatory process in the parapharyngeal or retropharyngeal area and a confirmatory CT were included in the study. 4 patients with severe complications (2 airway compromise and 2 mediastinitis) that required an urgent surgery, without the possibility of conservative treatment, were excluded. The criteria for deciding whether a patient could not be treated conservatively were: airway compromise, poor response to antibiotic treatment, immunocompromised patients or patients with associated complications (mediastinitis, internal jugular thrombophlebitis, involvement of lower cranial nerves…). All patients included in the study for conservative treatment were clinically stable at the time of admission, so none were considered candidates for intensive care unit monitoring after being examined by a pediatrician. Information was obtained regarding age, sex, presenting symptoms, physical examination, laboratory and imaging evaluations, management, duration of hospital stay and complications. Only seven parents (23%) did not consult previously to a primary care physician. During admission all patients were treated with intravenous amoxicillin/clavulanic acid; all of them were treated with corticosteroids (methylprednisolone). The patients were discharged after complete resolution of the clinical signs: resolution of torticollis and fever, analytical improvement, proper oral tolerance and after being evaluated by a pediatrician. None of the patients underwent a follow-up CT at the end of the treatment. None of the patients had comorbidities. The statistical analysis was performed with SPSS statistical software (version 19.0 for windows; SPSS INC, Chicago, Ill). The Student’s *t*-distribution was used for comparison of the data. The significance level was *p* < 0.05.

## Results

30 cases of RPA and PPA treated in our institution over a 10-years period were reviewed (11 RPA and 19 PPA). 18 patients (60%) were boys and 12 (40%) were girls. Age, initial symptom at presentation, and symptoms at diagnosis are shown in Table 1. Patients ranged in age from 1 to 14 years with a mean age of 4.6 years (95% CI 3.43–5.83 years); 76.7% of them were younger than 5 years old. Torticollis and fever were present in all patients, odynophagia in six patients (20%), only one patient presented with trismus (3.3%). The most common initial symptom was fever (83.3% of the cases). The mean time between the initial symptoms and the admission at the emergency department was 3.6 days (95% CI 2.69–4.71 days) (range 0.5–15 days), and 23 patients (76.7%) were treated previously by a primary care physician (seven received oral antibiotics and 16 an anti-inflammatory drug). None of them had a history of adenoids or tonsils surgery. The most common physical findings at admission were torticollis (100%), lymphadenopathy (96.7%) and hyperemic tonsils (76.7%). Only five patients (23%) showed medial displacement of the lateral oropharyngeal wall.

**Table 1:** Age, initial symptom at presentation, and symptoms at diagnosis of the children

Patient	Age	Initial symptom	Torticollis	Fever	Odynophagia	Trismus	Headache
1	6	Fever	Yes	Yes	Yes	No	No
2	3	Fever	Yes	Yes	Yes	No	No
3	3	Fever	Yes	Yes	Yes	No	No
4	4	Fever	Yes	Yes	Yes	No	No
5	4	Headache	Yes	Yes	No	No	Yes
6	12	Odynophagia	Yes	Yes	Yes	Yes	No
7	4	Odynophagia	Yes	Yes	Yes	No	No
8	3	Fever	Yes	Yes	No	No	No
9	4	Fever	Yes	Yes	No	No	No
10	3	Fever	Yes	Yes	Yes	No	No
11	1	Fever	Yes	Yes	Yes	No	No
12	2	Fever	Yes	Yes	Yes	No	No
13	3	Fever	Yes	Yes	No	No	Yes
14	6	Headache	Yes	Yes	Yes	No	No
15	2	Fever	Yes	Yes	Yes	No	No
16	5	Fever	Yes	Yes	Yes	No	No
17	5	Fever	Yes	Yes	No	No	Yes
18	14	Headache	Yes	Yes	No	No	No
19	2	Fever	Yes	Yes	Yes	No	No
20	11	Fever	Yes	Yes	Yes	No	No
21	3	Fever	Yes	Yes	Yes	No	No
22	5	Fever	Yes	Yes	Yes	No	No
23	1	Fever	Yes	Yes	Yes	No	No
24	8	Fever	Yes	Yes	Yes	No	No
25	2	Fever	Yes	Yes	Yes	No	No
26	11	Fever	Yes	Yes	Yes	No	No
27	4	Fever	Yes	Yes	Yes	No	No
28	2	Fever	Yes	Yes	Yes	No	No
29	4	Fever	Yes	Yes	Yes	No	No
30	2	Fever	Yes	Yes	Yes	No	No

**Fig. 1:**
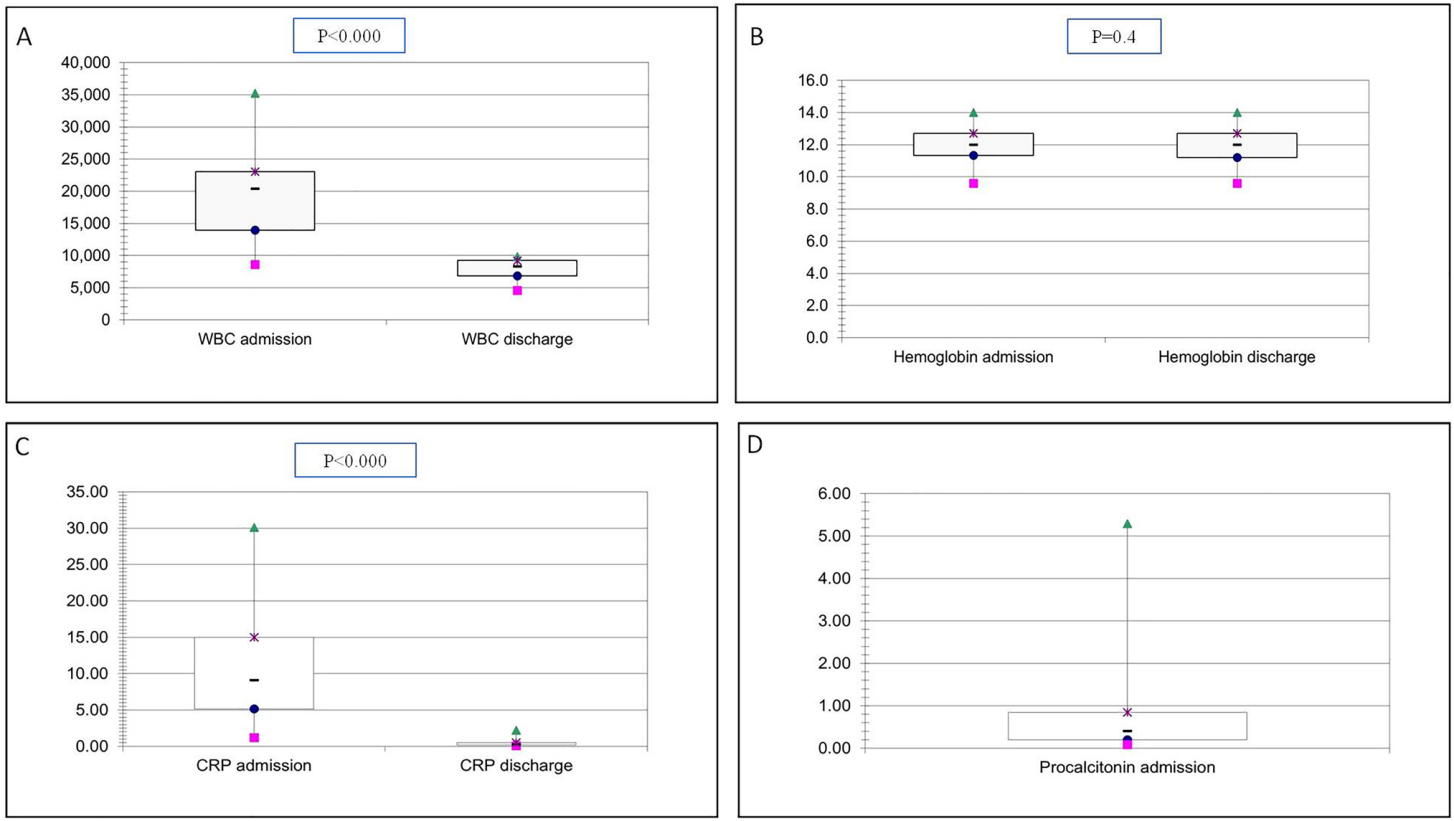
Boxplot diagrams for WBC (**A**), Hemoglobin (**B**) and CRP (**C**) at admission and discharge, and for Procalcitonin (**D**) at admission

White blood cell (WBC) counts were available in all patients, and all of them had leukocytosis, with a mean value of 20.045/µL (95% CI 17.420–22.671.4/µL) (range 8.590–35.210/µL) (reference value 5.000–10.0 00/µL). Mean hemoglobin value was 11.9 g/dL (95% CI 11.54–12.26 g/dL) (range 9.6–14 g/dL) (reference value 11.1–14.1 g/dL). C-reactive protein (CRP) value was also available in the 30 patients at admission, and its level was increased in all of them, with a mean of 11.4 mg/dL (95% CI 8.5–14.3 mg/dL) (range 1.2–30.1 mg/dL) (reference value 0.0–0.5 mg/dL). Procalcitonin was tested in only 22 of the 30 patients at admission (73.3%), with a mean of 0.87 ng/mL (95% CI 0.34–1.4 ng/mL) (range 0.08–5.29 ng/mL) (reference value 0.0–0.5 ng/mL). At discharge, none of the 30 patients had leukocytosis, mean WBC count of 8.008/µL (95% CI 7.086.1–8.929.9/µL) (range 4.560–9.800/µL). Mean hemoglobin value was 11.7 g/dL (95% CI 11.37–12.03 g/dL) (range 10–13.8 g/dL). CRP levels were obtained for 29 patients at discharge, and the mean value was 0.48 mg/dL (95% CI 0.32–0.64 mg/dL) (range 0.1–2.19 mg/dL). Differences between WBC at admission and discharge reached statistical significance (*p* < 0.000), as well as differences in CRP (*p* < 0.000). Hemoglobin levels were very similar between admission and discharge without statistical significance (*p* = 0.4). Analytical findings are shown in Fig. 1 as boxplot diagrams, showing the analytical improvement of WBC and CRP.

**Fig. 2:**
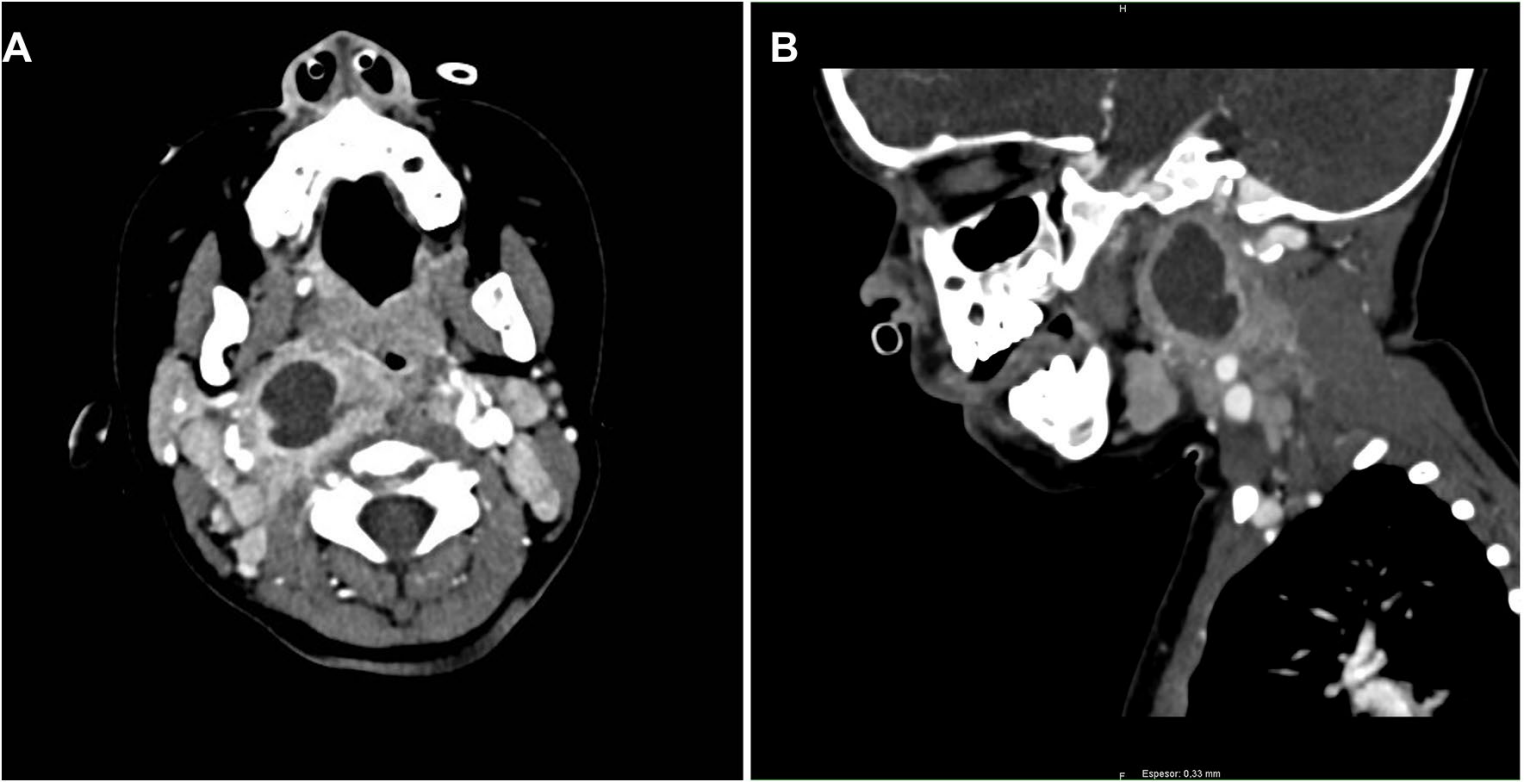
**A** Axial contrast enhanced CT scan of a 2-year-old patient showing a sharply demarcated lucency (arrow) in the right PPS (19.6 × 17.3 mm). **B** Sagittal contrast enhanced CT scan of the same patient

CT scan was performed in all patients at admission to confirm the diagnosis; all the cases showed a lucency located in the parapharyngeal or retropharyngeal space with ring enhancement, with a mean diameter of 25.7 mm (95% CI 22.2–29.2 mm) (range 12–50 mm) (Fig. 2). All patients were treated with intravenous amoxicillin/clavulanic acid (100 mg/kg per day) with corticosteroids associated in all of them, we used methylprednisolone (1 mg/kg per 12–24 h). Corticosteroids were used for 2–3 days (mean 2.7 days, 95% CI 2.53–2.87 days). All patients showed a favorable evolution with the conservative treatment and none of them needed surgery. There were no complications, and the mean length of stay was 7.5 days (95% CI 5.9–9.1 days) (range 3–22 days). At discharge, oral antibiotic treatment (27 amoxicillin/clavulanic acid and 3 cefuroxime) and oral anti-inflammatory on demand was prescribed to all patients.

## Discussion

DNIs are serious and occasionally life-threatening complications of nasopharynx or oropharynx infections and remain an important clinical entity in childhood. These conditions have increased their incidence in the last decades, in part due to the greater use of antibiotics that contribute to an increase in drug-resistances. Treatment of children diagnosed with DNIs is evolving, but still remains a significant debate about its treatment, with authors who advocate early surgical intervention with pus drainage and authors who suggest a medical treatment [12, 13]. DNIs can cause serious complications such as mediastinitis, acute airway obstruction, etc. [1]. The incidence of complications is still unclear and varies between studies. Baldassari et al. found a 9.4% incidence of complications, being the most common one mediastinitis, followed by airway obstruction necessitating intubation, persistent disease requiring repeat drainage and jugular vein thrombosis; in their study, several children experienced more than one complication, including four children who had both airway obstruction and mediastinitis [14]. No complications occurred during the treatment among our population.

In our study, DNIs were more frequent in younger children, especially in those who were 5 years old or less. This age incidence is similar to other series, and it is probably due to the immaturity of the immune system in them; in fact, lymph nodes in the retropharyngeal space usually disappear after 5 years. We found a predominance of boys in our study (60%), although the reason for this predilection is not clear.

Surgical drainage with intravenous antibiotic treatment was the traditional mainstay of management of pediatric DNIs, but more recently numerous investigators have advocated to a more conservative approach [1–4, 6, 11, 15]. Surgical drainage for PPA is generally performed through a cervical incision in the upper part of the neck or in the submandibular area. Injury to cranial nerves VII, IX, X, XI, or XII or to great vessels (internal jugular vein; common, internal, or external carotid arteries) can occur during surgery [4]. Also, this surgery causes a visible scar in the neck with a liability for local complications such as hypertrophic scars or keloid. For RPA, surgical drainage is usually performed through an intraoral approach, which avoids scarring on the neck, but it has other possible complications such as lesion of the great vessels, which can be difficult to control through this approach or impossibility to reach the abscess, especially in those of superior location [1]. Regarding PPA, in a recent study [16] that included 1174 patients divided into two groups according to whether they were treated with transoral or transcervical drainage, it was observed that there was no significant difference in the reduction of postoperative complications between transoral and cervical drainage; furthermore, transoral drainage appears to be feasible only in cases of abscesses that are localized and single in site.

Due to this fact, some authors [15, 17] advocate for intravenous antibiotic alone and surgery reserved for those who did not improve after several days or developed complications. Wilkie et al. [15] included 93 children, 15 of them underwent immediate surgical drainage because of life-threatening complications. Of the remaining 78 children who were managed conservatively in the first instance, 42 underwent delayed surgical drainage, and the remainder (*n* = 36) were successfully managed in a conservative way. Cheng et al. [17] included 178 children, with 159 initially treated with parenteral antibiotic therapy; children in the medical management cohort were successfully managed without surgery in a 66.3%. In 41 (23.0%), the patients’ clinical symptoms did not improve after a minimum of 24 h (including persistent fever, ongoing pain, trismus, or neck stiffness). In some cases, imaging studies were repeated, due to patient’s failure to clinically improve or progression of the infection, the patient eventually underwent delayed surgical drainage. In our series, all patients were treated medically with no need of surgery, there were no complications and none of them had an extra TC during the admission.

Wong et al. [18] included 54 children in their study, with half of them having abscesses ≥ 25 mm of maximum diameter. In the smaller size group (< 25 mm), 13 patients (52%) were successfully treated with antibiotics alone, with no need of surgery. On the other hand, in the bigger size group (> 25 mm), only four patients (15%) responded to antibiotics and were managed conservatively, while the rest underwent surgery. They concluded that high dose intravenous antibiotics may be an effective treatment for DNIs and may obviate the need for surgical drainage in smaller abscesses. Furthermore, two previous larger studies, one US series [17] that included 178 children with DNI and one European series [19] that included 101 children with RPA, showed that abscesses sizes of > 2.2 cm and > 2.0 cm, respectively correlated with requirement for delayed surgical drainage following an initial trial of medical management. In all these series, the medical management consisted on parenteral antibiotic therapy with no corticosteroid therapy associated. In our series, 16 patients had abscesses ≥ 25 mm, but all of them were successfully managed conservatively with antibiotic plus corticosteroid therapy regardless of the size. Therefore, although in other studies the size of the collection seems to be a determining factor for the success of medical treatment, in our study all patients, even those with abscesses with diameters greater than 25 mm presented a satisfactory evolution. In our opinion, corticosteroid treatment helps patients’ symptoms to resolve earlier, improving the general condition of the children.

Controversy exists on the use of steroids in the realm of DNIs due to their immunosuppressive effects. In our series, we treated our 30 patients with intravenous antibiotic and with corticosteroid therapy for 48–72 h, which conduced to a great improvement of symptoms (especially torticollis and odynophagia) in shorter than 24 h. Amoxicillin–clavulanic acid was chosen because of the relatively high percentage of anaerobic bacteria reported in the literature [3]. There is a paucity of literature on the use of corticosteroids as an adjunctive treatment to antibiotic therapy in DNIs. Tansey et al. [20] included 153 patients with DNIs; all of them received antibiotics and 53 (34.6%) received dexamethasone. The rate of surgical drainage in the dexamethasone and non-dexamethasone group was 36% and 53% respectively (*p* = 0.043). Furthermore, the mean length of stay was shorter for the dexamethasone group (2.9 days) compared to the non-dexamethasone group (3.8 days) but was non-significant. Therefore, we suggest that corticosteroids may produce a synergistic effect on treating abscesses that lead to better outcomes. DNIs could be treated with intravenous antibiotic and corticosteroid, with safe and excellent results, and this could help to reduce the length of stay, being in our series of 7.5 days, and to achieve an earlier improvement of most of symptoms.

DNIs are usually developed as a complication of a previous adenoiditis or tonsillitis, so it`s reasonable to believe that adenoidectomy and tonsillectomy may be protective; it would be consistent with the fact that none of the patients in our series had been adenoidectomized or tonsillectomized. On the contrary, some authors defend that adenotonsillectomy may be associated with the development of DNIs because of the decrease in the level of immunoglobulins following tonsillectomy [21]. In a recent study, Kim et al. [22] included 5.695 participants who underwent a tonsillectomy, classified into 5-year intervals; they found that DNIs were 1.43 times more frequent in the tonsillectomy group than in the control group. In subgroup analysis of adolescents and adults, this ratio was 1.87 times higher in the tonsillectomy group, but this risk did not increase in children. Kaygusuz et al. [23] found that tonsillectomy does not compromise the immune function of children during short-term (3 months) and long-term (54 months) follow-up. Therefore, evidence in support of the hypothesis that a decrease in immune function after tonsillectomy may increase the risk of deep neck infection is still lacking.

Our study found the most common presenting symptoms to be torticollis and fever; odynophagia was also present in more than three fourths of the children. These symptoms should be the clinical clue to diagnosing DNIs in children, and they can take to an early diagnosis and treatment, preventing serious complications. Most children had an upper respiratory tract infection in their histories, and parents wait for a mean time of 3.6 days, to consult a physician, usually when the torticollis appeared. Therefore, torticollis is the symptom of concern for parents that caused them to consult a physician.

A satisfactory physical examination it is not usually easy to achieve in children, so image studies are important in the diagnosis of DNIs. Haug et al. [24] established some rules that are important to detect alterations in para or retropharyngeal space with a lateral neck radiograph (adequate lateral orientation, correct extension of the neck and full inspiration); because of the short age of the patients and the impairment in the general status, it is difficult to comply them. Furthermore, after reporting an 83% sensitivity compared with a 100% sensitivity of contrast enhanced CT imaging, Nagy and Backstrom [25] advised against the use of lateral neck radiographs in the diagnostic work up of DNI. In our study, we performed an urgent CT scan on the day of admission to confirm the suspicious diagnosis. This technique is the current imaging study of choice for this purpose [26] and it is helpful in diagnosing and assessing the extent of infections of the retropharyngeal and parapharyngeal spaces. In an attempt to avoid exposure to ionizing radiation, the use of magnetic resonance imaging (MRI) is being analyzed. In the study by Conte et al. they observed that CT and MRI showed similar accuracy in predicting successful pus drainage during surgery [27]. Zhao et al. described the use of CT and ultrasound (US) imaging for the evaluation of neck infections in pediatric patients, and they concluded that children with neck infections evaluated in general emergency departments are significantly more likely to undergo CT scans when compared to those evaluated by pediatricians [28]. Despite MRI having the benefits of no radiation and a predictive positive value as high as 95% [29] it is infrequently used to evaluate uncomplicated DNIs in children. This may be secondary to the higher cost of MRI imaging or the need to sedate younger children to complete the exam, that could amplify the risk of evolving airway obstruction. On the other hand, US has been shown to be useful in differentiating peritonsillar abscess from other tonsillar infections. It has the advantage of being a quick, noninvasive, painless, cost-effective, and easily available technique, in addition to not requiring ionizing radiation or sedation. It can be useful to differentiate an isolated involvement of the tonsillar space from a DNIs, but if the latter is suspected, CT scan will be more reliable to identify concomitant spread of infection to other neck spaces [30, 31]. In our institution, the possibility of an urgent MRI is very limited and sometimes the upper location of the PPA or RPA means that ultrasound does not diagnose them, having to perform a CT if suspected.

CRP is an acute phase reactant whose value is usually elevated in this kind of infections. Pulliam et al. [32] observed that a CRP value higher than 9 mg/dL was related with a 67% probability of severe bacterial infection. In our series, we reported a mean value of 11.4 mg/dL in this parameter. In our study, the WBC mean value was 20.045/µL. Rzepakowska et al. [33] found that a leukocyte count over 11.000/µL was a significant predictor of prolonged hospitalization but did not increase complication rates. The same author found that hemoglobin levels lower than 12.5 g/dL was a prognostic variable of prolonged hospitalization; in our series the mean value for hemoglobin was 11.9 g/dL. Procalcitonin is a prohormone of calcitonin; inflammatory processes induce extra-thyroid production of procalcitonin, which is almost undetectable under physiologic conditions. Several studies [32–35] have reported that procalcitonin may be useful for early diagnosis as an indicator of severity in children with sepsis. Fioretto et al. [35] suggest that levels of procalcitonin between 0.5 and 2 ng/mL indicates a possible sepsis; in our series, the mean value for procalcitonin was 0.87 ng/mL, but we only obtained this value in 22 of the 30 patients (76.7%).

## Conclusions

DNIs represent dangerous entities with very serious potential complications. Increased attention should be placed on the typical neck examination; torticollis is the most important symptom to get early diagnoses. In a stable patient, intravenous antibiotic treatment with corticosteroids is a good and safe alternative to surgical drainage. Despite our good results, each case must be analyzed, alarm symptoms (airway compromise, lack of response to antibiotic therapy, immunocompromised patients…) and CT scan are the keys to decide between medical treatment or surgery.

## Abbreviations

DNIs: Deep neck space infections
RPA: Retropharyngeal abscesses
PPA: Parapharyngeal abscesses
MRSA: Methicillin-resistant *Staphylococcus aureus*
CT: Computed tomography
MRI: Magnetic resonance imaging
US: Ultrasounds
WBC: White blood cell
CRP: C-reactive protein
